# Prevalence of traditional eye medicine and self-treatment in Gurage Zone, Rural Ethiopia

**DOI:** 10.1186/s12906-024-04565-4

**Published:** 2024-07-04

**Authors:** Sadik Taju Sherief, Munira Sherefedin Sitotaw, Abonesh Girma

**Affiliations:** 1 Department of Ophthalmology School of Medicine, College of Health Sciences Addis Ababa University, P.O. Box 9086, Addis Ababa, Ethiopia; 2 York University, Toronto, Canada; 3 Niser Eye Clinic, Addis Ababa, Ethiopia

**Keywords:** Traditional eye medicine, Self-treatment, Rural community, Health seeking behavior, Sub-Saharan Africa

## Abstract

**Introduction:**

Traditional medicines are commonly used worldwide, especially in Africa—however, there is limited information on the prevalence and types of traditional eye medicine utilization in Ethiopia. The goal of this study was to determine the prevalence, the type and nature of traditional eye medicine use and practices related to self-medication for ophthalmic diseases in a rural Ethiopian population.

**Methods:**

A cross-sectional study was conducted in six randomly selected primary health centers in rural Gurage Zone, Southern Ethiopia. Health-seeking behavior, use of self-medication, and traditional eye medicine were assessed in the population using a semi-structured questionnaire.

Descriptive statistics and multivariable logistic regression analysis were computed to determine associated factors for using self-medication and traditional eye medicine.

**Result:**

Of the 814 participants interviewed, 487 (59.8%) reported using traditional eye medicine, mainly for combinations of symptoms of ocular redness, irritation, and eye discharge (95.5%). Besides, 604 (74.2%) participants reported self-treatment with tetracycline 1% eye ointment. Older age, females, low income, no formal education, and lack of access to media were risks for utilizing traditional eye medicine.

**Conclusion:**

The use of traditional eye medicine and self-treatment are common in this population. Regulatory legislation, public awareness, and making eye care are vital activities required to monitor such practices.

## Introduction

The World Health Organization (WHO) defines traditional medicine as “health practices, approaches, knowledge and beliefs incorporating plant, animal and mineral-based medicines, spiritual therapies, manual techniques, and exercises, applied singularly or in combination to maintain well-being, as well as to treat, diagnose or prevent illness.” [1]. According to a WHO survey, between 70 and 80% of the world's population uses non-conventional medications, many of which are derived from herbal sources [2]. Traditional eye medicines (TEM) are biologically based therapies or practices that are instilled or applied to the eye or administered orally to achieve a desired ocular therapy [3].

In Africa, Asia, and the Middle East, using TEM to treat many eye conditions is a common practice [4–16]. The applications of TEM vary across the nations; in India, alum water, milk, plant juices, saline water, breast milk, turmeric, jaggery, curd, garlic, goat’s milk, “neem,” the powdered horn of deer, excreta of a donkey, lemon juice, turpentine oil, coconut oil, warm tea leaves, ginger juice, onion juice, ash of Hanukkah, mustard oil, fenugreek, carom seeds, and leaf extracts are commonly used as TEM [17]. In Oman, Honey is used to alleviate ocular pain [15]; in Saudi Arabia, Alum*, *Calotropis procera, Aloe vera, and honey are commonly used TEM [16].

Traditional eye practices can be harmless or harmful in their consequences. Traditional healers' incantations or face washing with water are harmless traditional eye practices. [18, 19]. Ocular instillation of herbal extracts, breast milk, ground cowberries, and bird and reptile excrement are among the harmful TEM [18, 19].

Poor visual, ocular, and occasionally survival outcome of normally treatable eye disorders have been linked to use of TEM, whether as the only first-line treatment or as adjunct therapy used in conjunction with conventional therapy [20–23]. In developing countries, the inappropriate use of TEM increased the burden of infectious keratitis [7, 24, 25].

In Africa, TEMs are responsible for an estimated 8–10% of corneal blindness [26]. According to a study from Nigeria, 30% of endophthalmitis cases were caused by using TEM [27]. A study conducted in Tanzania showed that 25% of corneal ulcers resulted from using TEM [28]. In rural Malawi, 33% of patients with corneal disease reported having used TEM [21]. Numerous studies have established inconsistent associations between TEM use and socio-demographic variables [6, 15, 24, 29, 30]. Self-administered eye drops for ophthalmic conditions are also common practice in rural populations [31].

Nearly 80% of the population in Ethiopia relies heavily on herbal treatments as their primary source of therapy, and TEM use is a large carried out practice all over the country [32, 33]. A population-based survey conducted in Southern Ethiopia showed that 90% of the population uses TM in Ethiopia [34].

Information on the prevalence and pattern of utilization of TEM is scarce. A hospital-based study in Northern Ethiopia revealed that 22.31% of patients used TEM within two years of their presentation at an outpatient department of the referral hospital [12]. Though traditional medicine is a common practice in Ethiopia, the prevalence and pattern of TEM utilization have yet to be studied in the Ethiopian population.

This community-based study aimed to determine the prevalence and determinants of the use of TEM and self treatment in rural communities in Southern Nations, Nationalities, and People region of Ethiopia.

## Methods

### Study design and settings

This health center-based cross-sectional study was conducted in the Gurage zone, located in the Southern nation’s nationalities and people’s regional state (SNNPR) of Ethiopia from Dec 2017 to February 2018. The zone has 13 districts, two town administrations, 412 rural and 32 urban kebeles and 22 health centers in the rural areas [35, 36]. The department of Ophthalmology of Addis Ababa University granted ethical approval. Before participating in this study, all subjects fully understood the purpose of the study and signed an informed consent form; for those children under 16 years, parents and/or guardians signed the informed consent form.

The sample size was calculated based on the single population proportion formula by using the formula (*n* = (zα/2)2 p (1-p)/ d2) for estimating a single population proportion at a 95% confidence interval, where p was the prevalence of TEM utilization in South Africa [37]. The calculated sample size with 15% non-response was used and multiplied by 2 for design effect; the final sample size became 814.

The sample was allocated proportionally for the six randomly selected health centers from six rural districts, and a systematic random sampling technique was employed to select individual patients from each of the outpatient clinics of the health centers.

### Data collection procedure and data quality control

The data collection tool was prepared after reviewing related literature [8, 10, 15, 24, 31, 37]. The questionnaire was pretested among 5% of patients with ocular diseases in a similar setting but outside the sampled health centers and found valid through the appropriate Cronbach’s alpha test (> 0.7).

Interviewer administered interview technique was used to collect data with structured and pretested questionnaire. All patients with ocular diseases who attended the outpatient clinics of the selected rural health centers were eligible for the study. For those patients aged less than 18 years, parents /guardians were interviewed. Four nurses collected data after training for a day and were supervised by the principal investigator. The questionnaire sought information on the following:

- Socio-demographic characteristics.
- History of eye disease in the past year.
- Utilization of TEM and self-medication.
- Types of TEM used.

The data collector and the principal investigator checked each questionnaire for completeness.

### Operational definition

*Traditional eye medicines (TEMs)*: are a form of biologically- based therapies or practices that are instilled or applied to the eye or administered orally to achieve a desired ocular therapeutic effect [3].

*Self-medication*: is the use of medicines without consulting a doctor by one’s initiative or on the advice of another person in the last two months [38].

### Statistical analyses

Basic descriptive statistical analysis was undertaken using IBM SPSS Statistics (Version 16.0). Associations between TEM and self-treatment and continuous and categorical variables were computed using Fisher’s exact and Pearson chi-square (χ2) tests, respectively. Continuous variables were compared using ANOVA. Values of *p* < 0.05 were considered statistically significant.

## Results

### Demographic characteristics of the study population

Eight hundred and fourteen patients were interviewed to evaluate TEM and self-treatment application practices. More females than males were interviewed (60% and 41.1%, respectively). The mean age of the study participants was 47.02 + 15.8 years (range 14–90 years). Twenty-one of the interviewed participants were children aged less than 14 years (shown in Table 1).

**Table 1:** Age by sex distribution of patients participated in the study, Dec 2017- Feb 2018

**Age Group**		**Male**	**Female**	**Total**
		n (%)	n (%)	n (%)
	< 18 years	11 (3.4)	10 (2)	21 (2.6)
	19–40 years	117 (35.9)	183 (37.5)	300 (36.9)
	41–60 years	123 (37.7)	232 (47.5)	355 (43.6)
	> 60 years	75 (23)	63 (12.9)	138 (16.9)
**Total**		326 (100)	488 (100)	814 (100)

Among the study participants, the majority (561, 68.9%) had no formal education, 333 (40.9%) of them were house workers, 609 (74.8%) earned less than 1,000 ETB per month, and 431(53%) of them were Muslim by religion (Table 2).

**Table 2:** Association of socio-demographic factors with use of traditional eye medicine in the study population

			**TEM USE**		**OR** **(95% CI)**	*P*-value	X^2^- value
**Variable**	**Category**	**Total** **n (%)**	**Yes (%) *n* = 487**	**No (%) *n* = 327**			
**Age**	< 18 years	21 (2.6)	4 (19)	17 (81)	0.15 (0.05–0.43)	0.0001	14.92
	> 18 years	793 (97.4)	483 (17)	310(83)			
**Gender**	Male	326 (40)	157(48.2)	169 (51.8)	0.44 (0.33–0.59)	0.0001	30.81
	Female	488 (60)	330 (67.6)	158( 32.4)			
**Religion**	Christian	383 (47)	198 (51.7)	185 (48.3)	0.86(0.65–1.13)	0.2835	1.15
	Muslim	431 (53)	239 (54.5)	192 (44.5)			
**Literacy Status**	No Formal education	561(68.9)	457 (84.5)	104 (16.5)	32.66 (21.11–50.55)	< 0.00001	351.48
	Formal Education	253 (31.1)	30 (11.9)	223 (88.1)			
**Occupation**	House work	333 (40.9)	280 (84)	53 (16)			
	Formal Work	481 (59.1)	207	274	6.99 (4.95–9.87)	< 0.0001	137.95
**Income per month**	< 2000 ETB	761 (93.5)	484 (63.6)	277 (36.4)	29.12 (8.99–94.24)	< 0.0001	69.21
	> 2000 ETB	53 (6.5)	3 (5.7)	50 (94.3)			
**Access to Media**	Yes	449 (55.2)	148 (33)	301 (67)	0.04(0.02–0.06)	< 0.0001	300.71
	No	365 (44.8)	339 (92.9)	26 (7.1)			

### Utilization of healthcare services

Seven hundred and fifty-four (92.6%) subjects had ocular complaints within the prior year. Of these, 418(50.8%) visited nearby health institutions, 254 (30.9%) had home treatment, and 82 subjects (10.9%) visited traditional health practitioners. All subjects who visited THs were given herbal medicines, and 15 of them additionally received animal products of traditional medicine. Direct instillation of the herbals' juice was the commonest medication administration route. One hundred and eight patients (13.6%) of the total study subjects visited traditional health practitioners at least once in their lifetime for their ocular complaints.

### Use of traditional eye medicine

Of the 814 participants asked about using any traditional product in their eyes within one year from the interview date, 487 (59.8%) mentioned using TEM for various ocular complaints. A combination of ocular redness, irritation, and eye discharge was the main indication (*n* = 465; 95.5%) to use the TEM.

**Fig. 1:**
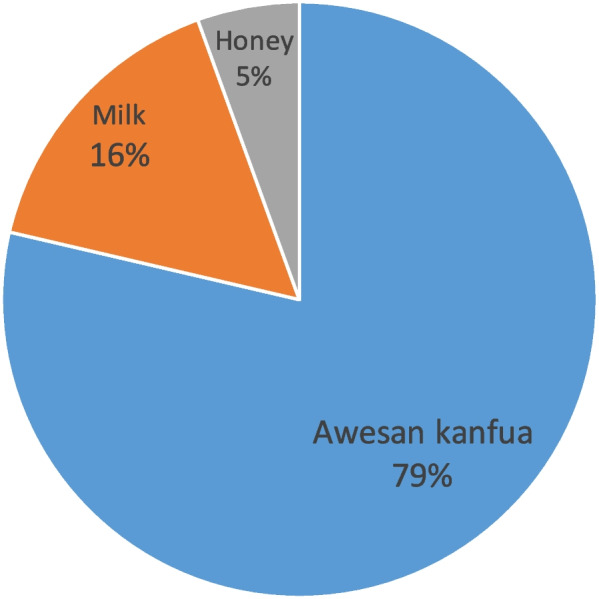
Types of traditional eye medicines used by the study population. n- 487, types of traditional eye medicine used by the study population

The traditional eye medicines commonly used in this study group (*n* = 487) were “Awesan kanfua” (Cyathula uncinulata) ( *n* = 383; 78.6%), milk (*n* = 77; 15.8%), and honey (*n* = 27; 5.5%) (Fig. 1). Cyathula uncinulata is a herbal that is utilized by massaging the eye. Cyathula uncinulata and milk were used for ocular redness, discharge, and irritations, while honey was used for poor vision. Cyathula uncinulata and milk were directly applied to the eye, whereas honey was applied by rubbing the eyelids.

Distant location of the eye care facilities (178/487, 36.5%), recommendations from older people (159/487, 32.6%), and a strong belief in traditional treatment (89/487, 21.1%) were the main reasons for using TEM.

Traditional eye medicine utilization was found to be more common among elderly subjects aged above 60 years (85.5%), females (67.6%), no formal education (84.5%), housewives (84%), monthly income less than 1000 ETB and respondents with no access to eye health care (92.9%). Traditional eye medicine utilization was reported to be statistically significant in adults ( X^2^ = 14.92, *p* = 0.0001), females (X^2^ = 30.81, *p* = 0.0001), not attending formal education (X^2^ = 351.58, *p* = 0.00001), housework (X^2^137.95, *p* < 0.00001), less monthly income (X^2^ = 69.21, *p* < 0.0001), and no access to media (X^2^ = 300.71, *p* < 0.0001) (Table 2).

### Self-treatment

Nearly three fourth (*n* = 604; 74.2%) of the study population interviewed reported the use of ophthalmic medicines without consulting health professionals for symptoms of redness with eye discharge (*n* = 217; 35.9%), itching and redness (*n* = 109; 18%), watering (*n* = 97;16.1%), redness and burning (*n* = 83; 13.7%), painful eyes (*n* = 35; 5.8%), redness and foreign body sensation (*n* = 33; 5.5%), and burning of the eyes (*n* = 30; 5%). Tetracycline 1% eye ointment is the medication used by all respondents.

Self-treatment was found to be more common among elderly subjects aged above 60 years (87.7%), no formal education (89.6%), housewives (93.7%), and respondents with no access to eye health care (93.7%). Being not formally educated (X^2^ = 210.04, *p* < 0.001), house worker (X^2^ = 111.85, *p* < 0.001), less monthly income (X^2^ = 6.21,* p* = 0.0123) and no access to media (X^2^ = 131.4, *p* < 0.001) subjects were more likely to self-medicate (Table 3).

**Table 3:** Association of socio-demographic factors with self–treatment with eye medications in the study population

			**Self Treatment**		**OR** **(95% CI)**	*P*-value	X^2^- value
**Variable**	**Category**	**Total**	**Yes (%) *n* = 487**	**No (%) *n* = 327**			
**Age**	< 18 years	21 (2.6)	13 (61.9)	8 (81)	0.55 (0.23–1.36)	0.192	1.7
	> 18 years	793 (97.4)	591 (17)	202(83)			
**Gender**	Male	326 (40)	241 (74)	85 ( 26)	0.98 (0.71–1.34)	0.02	0.89
	Female	488 (60)	363 (74.4)	125 (25.6)			
**Religion**	Christian	383 (47)	297 (77.6)	86 (22.4)	1.39 (1.02–1.92)	0.04	4.23
	Muslim	431 (53)	307 (71.2)	124 (28.8)			
**Literacy Status**	No Formal education	561(68.9)	500 ( 89.6)	61(10.4)	11.74 (8.15–16.92)	< 0.001	210.04
	Formal Education	253 (31.1)	104 (41.1)	149 (58.9)			
**Occupation**	House work	333 (40.9)	312 (93.7)	21 (6.3)	9.62 (5.96–15.51)	< 0.001	111.85
	Formal Work	481 (59.1)	292 (60.7)	189 (39.3)			
**Income per month**	< 2000 ETB	761 (93.5)	557 (73.2)	204(26.8)	0.35(0.15–0.83)	0.0123	6.21
	> 2000 ETB	53 (6.5)	47 (88.7)	6 (11.3)			
**Access to Media**	Yes	449 (55.2)	262 ( 58.4)	187 (41.6)	0.094 (0.06–0.15)	< 0.001	131.4
	No	365 (44.8)	342 (93.7)	23 ( 6.3)			

## Discussion

Many studies showed that TEM utilization is recognized as one of the important causes of corneal blindness and delay in presentation, especially in rural populations in developing countries [6, 7, 24, 25, 28]. To our knowledge, no study has been conducted in a rural setting on utilizing TEM and self-medication in Ethiopia. Our study highlights that the rural Ethiopian population utilizes traditional, home-based remedies and self-prescribed ophthalmic medications to relieve ocular symptoms. Similar to our research, the majority of studies on the use of TEM were conducted in rural areas and by health organizations.

The prevalence of the utilization of TEM in various studies ranges from 5.9% [39] to 82.3% [40]. The wide gap in the prevalence of TEM utilization among the various studies can be attributed to the difference in timing, design, study participants, setting, and socio-demographic variables. In our study, the prevalence of the use of TEM among those who have had eye disease in the past year is quite high (59.8%) in comparison with studies from the Democratic Republic of Congo (17.9%, [41], rural India ( 25%, [13], rural Malawi (33.8%) [6], South Africa ( 36%) [37], Uganda (44.2%, [42], semi-urban South Nigeria (48.7%, [11] and semi-urban Tanzania ( 49%) [30], but comparable with hospital-based studies from Zimbabwe ( 61.5%) [43] and Saudi Arabia ( 54.2%) [44]. However, our finding is lower than the study reported from South East Nigeria (82.3%) [40]. The higher incidence of TEM use in our study can be related to the community's strong faith in traditional medicine and the shortage of nearby eye care facilities, which was reflected in the reasons for choosing TEM.

In our study, herbal extract Cyathula uncinulata was known and used by most of the respondents. This finding is similar to other African studies [6, 11, 27, 39, 45]. But it is in contrast to a study in a South Indian hospital [24], where human breast milk was the most common form of TEM, a hospital-based study from Brazil [46], where homemade traditional products like boric acid, normal saline, and herbal infusions commonly used, and in Saudi Arabia and Oman where “Zemzem” holy water is the most widely used TEM [15, 46].

Honey was used to treat various Ophthalmic diseases like blepharitis, keratitis, post-herpetic corneal opacities, corneal edema, corneal injuries, and chemical and thermal burns [15, 44, 47]. Though we didn’t evaluate the efficiency, some respondents in our study mentioned they use honey to treat cloudy vision from cataracts.

Our study showed that the use of TEM was significantly statistically (*p* < 0.001) associated with being female, older, having lower income, being no formal education, and lacking access to media. This study found that females are more likely to use TEM than males (*P* < 0.001); it agrees with a hospital-based study from Saudi Arabia [44] and an eye clinic finding from the USA [48]. On the contrary, a study from a semi-urban community in Nigeria showed males were more likely to use TEM [11]. However, no significant gender differences were reported from studies in rural India [13] and Southeast Nigeria [39]. TEM use by females can have been attributed to gender-related barriers to accessing eye care services in rural Ethiopia.

No formal education, low-income participants and those who didn't have access to media were observed to have a significantly higher likelihood to use TEM than their better-educated, better income and with access to media counterparts. A significant association between low educational status and the use of TEM agrees with studies from Saudi Arabia [49] and South Nigeria [10]. Similarly, in studies from Niger [45], the Democratic Republic of Congo [41], and Nigeria [11], respondents with no formal education were more likely to use TEM than those with tertiary education. Our study was conducted in a rural setting with limited access to eye care. And the higher tendency to use TEM among the no formal educated, less access to media, and poor income show that geographic access and affordability are potential barriers to seeking modern eye facilities.

Regarding the use of TEM, various studies reported a large scale of ocular conditions like ocular trauma, corneal ulcers, redness, and irritations [6, 8, 23, 28]. This study has shown that most participants use TEM for ocular redness, irritation, and eye discharge. In the Edo State of Nigeria [11], the University of Benin [23], Nigeria, and Tanzania [30], poor vision was the most common reason for necessitating the use of TEM, Whereas, in Northern Ethiopia [12] and South-eastern Nigeria [39], most participants mentioned ocular trauma as an indication to use TEM. A corneal ulcer was the leading indication to use TEM in rural Malawi in South India and Nepal [6, 24].

The prevalence of active trachoma in various districts of the Zone ranges from 2.3%- 42.6%. [50]. Most respondents have clinical features of active trachoma. Besides, trachoma has been endemic within the community for decades; hence using the available traditional medicine which the community has been using for a long period can be the possible explanation to use TEM for ocular redness, irritation, and discharge.

Studies have shown that the frequent use of TEMs is led by the common belief that anything herbal and traditional implies the absence of any adverse effect [14, 51]. This finding is also reflected in our study, where more than 50% of the participants mentioned that they strongly believe in TEMs, and the elderly community recommended TEM.

In our study population, self-treatment was reported by nearly three-fourths (74.2%) of the population. Similar rates of self-treatment have been reported in Southern Chile (75%) [52]. Our finding is higher than reports from rural India (18.4%) [24]. A meta-analysis study in Ethiopia showed that the pooled prevalence of self-medication was 44%. Poor supervision by the governmental drug regulation body can be the reason for higher self-medication in our study.

### Limitation

We couldn't observe the method of preparation of TEM or the route of administration due to methodological limitations. Besides, we didn't add clinical features of the study participant to evaluate the outcome of TEM utilization.

## Conclusion

The prevalence of TEM utilization and self-treatment in the rural community of Southern Ethiopia was higher. Being female, older age, low-income, no formal education, and lack of access to media were all significant risk factors for TEM Utilization.

Further studies are needed to evaluate the ocular outcome of TEM in the community.

Regulatory legislation and public awareness activities must be implemented to monitor the utilization of TEM and self-treatment. Besides, eye health facilities must be accessible to the rural community.

## Data Availability

All data generated or analysed during this study are included in this published article.

## Abbreviations

ETB: Ethiopian Birr
TEM: Traditional eye medicine
THs: Traditional healers
WHO: World Health Organization
